# Comment on “Proximal Myopathy due to m.5835G>A Mutation in Mitochondrial MT-TY Gene”

**DOI:** 10.1155/2019/3514894

**Published:** 2019-07-01

**Authors:** J. Finsterer, C. A. Scorza, A. C. Fiorini, F. A. Scorza

**Affiliations:** ^1^Krankenanstalt Rudolfstiftung, Messerli Institute, Vienna, Austria; ^2^Disciplina de Neurociência, Universidade Federal de São Paulo/Escola Paulista de Medicina (UNIFESP/EPM), São Paulo, Brazil; ^3^Programa de Estudos Pós-Graduados em Fonoaudiologia, Pontifícia Universidade Católica de São Paulo (PUC-SP), Brazil; ^4^Departamento de Fonoaudiologia, Escola Paulista de Medicina/Universidade Federal de São Paulo (EPM/UNIFESP), São Paulo, Brazil

With interest we read the article by Simoncini et al. about a 53-year-old female with asymptomatic mitochondrial myopathy due to the MT-TY (tRNA(Tyr)) variant m.5835G>A [1]. We have the following comments and concerns.

It is contradictory to state that “her personal history was unremarkable” and to report at the same time that she had “a two-year history of progressive lower limb weakness” [1]. Her history is definitively not unremarkable but remarkable for weakness, postural changes, occasional dysphagia, and elevated creatine-kinase [1]. The phenotype is different from that of the patient described by Kornblum et al. in 2008 who also carried the m.5835G>A variant but presented with chronic progressive external ophthalmoplegia but without limb muscle weakness or dysphagia [2].

The detected mutation is reported to have been heteroplasmic in the muscle. We should be informed about the exact figure of the heteroplasmy rate in muscle, about the muscle in which heteroplasmy was determined, and if muscle biopsy was taken from a clinically affected or unaffected muscle.

The family history of the patient is also crucial. Particularly we should know if the mother and children of the index case carried the mutation as well and if it was clinically manifesting or not. Since the phenotypic expression of a single mutation may vary between family members [2], a pedigree with the clinical manifestations of all first-degree relatives should be provided.

Since mitochondrial disorders (MID) are frequently multisystem disorders at onset or become multisystem disorders during the disease course, we should be informed if other organs than the muscle were clinically or subclinically affected or if the patient developed involvement of other organs than muscle during follow-up. Was dysphagia of the index patient attributed to involvement of the smooth muscle cells, to depression, to involvement of the cerebrum, or to involvement of the peripheral nerves?

Not only the six patients listed in table 1 of the paper carrying a tRNA(Tyr) mutation have been described so far but also patients reported by Liu et al. and Baranowsaka et al. [3, 4].

Mutations in mtDNA located tRNA genes go along with dysfunction of the respiratory chain [5]. Thus, we should know the results of the biochemical investigations of the muscle homogenate and if a single or multiple respiratory chain complexes were compromised.

Overall, this interesting case would be more meaningful if heteroplasmy rates were provided, if biochemical investigations were carried out, if all reported patients carrying a MT-TY mutation were discussed, and if first-degree relatives were investigated.
